# Curcumin Sustained Release with a Hybrid Chitosan-Silk Fibroin Nanofiber Containing Silver Nanoparticles as a Novel Highly Efficient Antibacterial Wound Dressing

**DOI:** 10.3390/nano12193426

**Published:** 2022-09-29

**Authors:** Parisa Heydari Foroushani, Erfan Rahmani, Iran Alemzadeh, Manouchehr Vossoughi, Mehrab Pourmadadi, Abbas Rahdar, Ana M. Díez-Pascual

**Affiliations:** 1 Department of Chemical Engineering, Biomedical and Bioenvironmental Research Center (BBRC), Sharif University of Technology, Tehran 14179-35840, Iran; 2 Department of Biotechnology, School of Chemical Engineering, College of Engineering, University of Tehran, Tehran 14179-35840, Iran; 3 Department of Biomedical Engineering, University of Delaware, Newark, DE 19713, USA; 4 Department of Physics, University of Zabol, Zabol 98613-35856, Iran; 5 Universidad de Alcalá, Facultad de Ciencias, Departamento de Química Analítica, Química Física e Ingeniería Química, Ctra. Madrid-Barcelona, Km. 33.6, 28805 Alcalá de Henares, Madrid, Spain

**Keywords:** chitosan, silk fibroin, silver nanoparticles, curcumin, electrospinning, wound dressing, healthcare, drug delivery, biomedical engineering

## Abstract

Drug loading in electrospun nanofibers has gained a lot of attention as a novel method for direct drug release in an injury site to accelerate wound healing. The present study deals with the fabrication of silk fibroin (SF)-chitosan (CS)-silver (Ag)-curcumin (CUR) nanofibers using the electrospinning method, which facilitates the pH-responsive release of CUR, accelerates wound healing, and improves mechanical properties. Response surface methodology (RSM) was used to investigate the effect of the solution parameters on the nanofiber diameter and morphology. The nanofibers were characterized via Fourier Transform Infrared Spectroscopy (FTIR), X-ray Diffraction (XRD), Scanning Electron Microscopy (SEM), zeta potential, and Dynamic Light Scattering (DLS). CS concentration plays a crucial role in the physical and mechanical properties of the nanofibers. Drug loading and entrapment efficiencies improved from 13 to 44% and 43 to 82%, respectively, after the incorporation of Ag nanoparticles. The application of CS hydrogel enabled a pH-responsive release of CUR under acid conditions. The Minimum Inhibitory Concentration (MIC) assay on *E. coli* and *S. aureus* bacteria showed that nanofibers with lower CS concentration cause stronger inhibitory effects on bacterial growth. The nanofibers do not have any toxic effect on cell culture, as revealed by in vitro wound healing test on NIH 3T3 fibroblasts.

## 1. Introduction

Over the last decade, the incidence of infections among people has skyrocketed alarmingly fast. The skin plays a pivotal role in protecting against diseases caused by threatening pathogens, such as bacteria and viruses. Infection associated with the secretion and persistent presence of microorganisms in the wound area is among the difficulties imputed to the wound healing process [1,2]. Therefore, the advancement of treatment methods is linked to the fabrication of wound dressings as a requisite to resolve problems faced in the wound healing process; to this end, the use of biomimetic wound dressing providing a microenvironment that meets biological requirements has attracted further attention recently [3,4]. In fact, the incorporation of various biomaterials and implementing methods for optimizing the composition and properties of fabricated wound dressing opens the way for establishing an ideal wound dressing for facilitating wound healing and resolving problems associated with conventional methods. There are various forms of wound dressing such as films, sponges, and micro or nanofibers, of which nanofibers seem the most promising because of their similarity to the extracellular matrix and high surface-to-volume ratio [5]. There is strong evidence regarding the cost-effectiveness, simplicity, and efficiency of using electrospinning as a nanofiber fabrication method [6,7]. The unique properties of polymeric nanofibers present electrospinning as an efficient technique for fabricating drug-loaded electrospun nanofibers. In detail, drug delivery systems based on polymeric nanofibers can limit or remove the negative and adverse side effects of drugs [8]. Moreover, compared to other methods, electrospun nanofibers composed of polymeric constituents have shown a reduction in the initial burst release of drugs [9].

Due to their promotion of cell proliferation, migration, and attachment, biopolymers have become increasingly popular for fabricating promising electrospun platforms for tissue engineering and wound dressing in recent years [10]. Natural polymers including chitosan (CS), fibrinogen, elastin, and collagen are biocompatible substances, closely resembling macromolecules in the body. Chitosan (poly-[1–4]-β-glucosamine, CS) is a naturally derived polysaccharide polymer achieved with the deacetylation of chitin. CS has been widely used for tissue engineering and wound dressing applications regarding its characteristics, i.e., high biodegradability, low toxicity, biocompatibility, high biodegradability, and pH-sensitivity [11,12]. These properties ensure the capability of CS as a good substance in biomedical applications such as drug delivery [13,14], wound dressing [15], tissue engineering [16], and biosensors [17]. Despite the discussed benefits of CS for various biomedical fields, disadvantages like poor mechanical stability [18] and high swelling at neutral pH due to its highly pH-sensitive feature [19] impede its widespread application. The addition of synthetic or natural polymers with slow degradability and high mechanical properties to CS hydrogel can effectively resolve the discussed problems and paves the way for its wider application in tissue engineering and wound dressing. Regarding the cross-linking of CS-based hydrogels, glutaraldehyde (GA) and glyoxal are the most widely employed [20]. We opted for GA in this research instead of glyoxal. In a recent study, Gupta et al. [21] reported that the more controlled release of centchroman from CS crosslinked with GA microspheres as compared to CS microspheres crosslinked with glyoxal. While the use of GA as a cross-linker has certain drawbacks, including changes in the fiber morphology and a high risk for toxicity when high concentrations of GA are used [22], GA-crosslinked hydrogel is a promising candidate for fabricating wound dressing and tissue engineering platforms with reduced cytotoxicity and a controllable shape when a suitable crosslinking method with optimum GA content is employed.

Among many natural-based polymers, silk fibroin (SF) is a protein-based polymer made from various insects and spider species [23]. SF-based fibers have been frequently used as surgical sutures [24]. The properties of these fibers include slow degradability, high tensile strength, and flexibility [25]. The stability of this family of fibrous proteins is due to high hydrogen bonding, low hydrophilicity, and a degree of crystallinity arising from β-sheets [26]. Being compatible with the body, SF nanofibers support binding, spreading, and proliferation of fibroblasts and bone marrow cells, as shown by related studies [27,28]. Notwithstanding the many advantages of employing SF-based nanofibers for tissue engineering and wound dressing, SF’s major drawback (poor hydrophilicity) may confine its widespread employment [29]. This fault may be remedied by adding CS hydrogel because of its high hydrophilicity, maintaining the acceptable level of moisture in the wound area, thus accelerating the wound healing process.

Various microbial agents including chemical antibiotics, herbal antibiotics, and metal nanoparticles have been recently studied in combination with wound dressing and tissue engineering platforms [30,31,32]. Due to the fact that antibiotics target a specific range of microorganisms and cause bacterial resistivity, metal nanoparticles and herbal compounds are better options for wound dressings. Today, attention has been drawn to nanofibers containing metal nanoparticles for their optical, electrical, catalytic, and antimicrobial properties [33,34]. Among many metal components, silver (Ag) is desirable to intensify antibacterial properties and can be used for infection and wound healing when it reaches the nanoscale [34]. Nano-sized Ag affects the metabolism, respiration, and reproduction of a wide range of microorganisms and shows acceptable mechanical and thermal stability. Ag nanoparticles are capable of killing more than 650 types of pathogenic microorganisms including *E. coli* and *S. aureus* bacteria [35]. Ag nanoparticles not only have antibacterial but also anti-odor properties to reduce the smell of wound infection [36]. Nevertheless, the effectual application of Ag nanoparticles can be restrained due to their concentration limits. For example, Ag nanoparticles with a concentration of 25 mg/L caused significant toxicity in rainbow trout gill fish cell line RT-W1 [37]. This problem can easily be remedied if a lower concentration of Ag nanoparticles is used, making Ag nanoparticles more useful in wound dressing.

Curcumin (CU), categorized primarily into the flavonoid group of polyphenols, is a lipophilic drug insoluble in water and soluble in solvents such as dimethyl sulfoxide, acetone, ethanol, and chloroform [38]. It has anti-oxidation, anti-inflammatory, antibacterial, anti-virus, and anti-cancer properties and can be applied for wound dressing either orally or topically [39]. Recent studies have shown the efficacy of CU as an antitumor agent on breast cancer cells (MCF-7) by a p53-dependent pathway [40]. Due to its ability to stimulate the production of the growth factors and cytokines involved in the wound healing process [41], CU is an attractive drug for healing wounds in this regard.

In this study, we determined the effect of different solution parameters (CS concentration, GA volume and ethanol volume) on the mean diameter of SF-CS-Ag-CUR nanofibers and their morphology using the Response Surface Methodology (RSM). For improving cell adhesion and proliferation, a facile one-step green method based on employing CS hydrogel as both a reducing and stabilizing agent without using harmful chemicals was applied for the formation of Ag nanoparticles. Chemical structure, physical and mechanical properties, degradation, antibacterial activity, as well as cytotoxicity of the electrospinning nanofibers were tested. Because of abnormalities of cells in bacteria-infected wounds, these sites have specificities like an acidic pH, which can be used as triggers for drug release. To investigate the pH-responsive release of CUR as the model drug from the fabricated nanofiber, the in vitro release study was performed. To the best of our knowledge, this is the first original investigation filling the existing gaps in wound dressing, providing the pH-responsive release of CUR and improving CUR loading and entrapment efficiencies for ameliorating the major problems of CUR, i.e., low solubility.

## 2. Materials and Methods

### 2.1. Materials

LMW chitosan (CS, 50–190 kDa, deacetylation degree of 75–85%), acetic acid, sodium carbonate (Na_2_CO_3_), lithium bromide (LiBr), phosphate buffer saline (PBS), polyethylene oxide (PEO, Mw = 900,000 g/mol), and silver nitrate (AgNO_3_) were supplied by Merck. Sigma-Aldrich (Burlington, MA, USA) provided fetal bovine serum (FBS), dimethylsulfoxide (DMSO), Dulbecco’s Modified Eagle’s Medium (DMEM) High Glucose, 3-(4, 5-dimethylthiazol-2-yl)-2, 5-diphenyl-tetrazolium bromide (MTT), and curcumin (CUR). The American Type Culture Collection (ATCC) provided NIH 3T3 fibroblasts cell lines. Penicillin/streptomycin and 0.25% (*w*/*v*) trypsin–0.1% (*w*/*v*) ethylenediaminetetraacetic acid (EDTA) were purchased from Solarbio (Beijing Solarbio Science and Technology, China). *Escherichia coli* (*E. coli*) (ATCC 87398) and *Staphylococcus aureus* (*S. aureus*) (ATCC 25923) were provided by the Microbiological Resources Centre, Iranian Research Organization for Science and Technology.

### 2.2. Preparation of SF Solution

SF was prepared according to the previously reported literature [42]. First, Na_2_CO_3_ was added to 2 L of boiling deionized (DI) water to reach a concentration of 0.02 M. Next, 5 g of Bombyx mori cocoon was added, and the resulting solution was boiled for 1 h. The silk cocoons were then rinsed with DI water three times in order to extract water-soluble sericin. The obtained solution was dried under laminar flow overnight. After drying, LiBr (10 M) was added to the solution. Next, the solution was carried into an oven. The oven was heated and maintained at 60 °C for 4 h. Then, the solution was poured into a dialysis bag which was immersed in 2 L of water and kept for three days to complete the removal of LiBr. It is worth noting that six replacements of the bath water were needed. Finally, SF was obtained by centrifugation at 4500 rpm, 20 min, and 4 °C and washing three times with DI water.

### 2.3. CS-Ag-CUR Solution Preparation

In the first stage, acetic acid was added to DI water and maintained at 50 °C for 15 min to reach 40% (*v*/*v*) before adding to CS. Next, different concentrations (5, 7, and 9% *w*/*v*) of CS were slowly added to the prepared solution by the use of a heater stirrer (60 °C, 300 rpm) to reach a homogeneous solution. Owing to the sensitivity of SF to the vortex, we selected the CS solution for the loading of the drug and Ag nanoparticles. Regarding the crosslinking of Ag ions, sodium borohydride (NaBH_4_) is the most widely used [43]. Various harmful effects on human health regarding the application of NaBH_4_ were reported in previous studies [44]. Patrícia Carapeto et al. [45] investigated the reaction mechanism of Ag ions by CS. They concluded that in the reduction of two Ag^+^ ions to Ag^0^ nanoparticles, one carbonyl group is produced, originating from the oxidation of alcohol and/or glycosidic groups in CS. In this regard, in order to reduce the prepared nanofibers’ toxicity, we only used CS as a reducing and stabilizing agent. Next, 10 mg of AgNO_3_ was added to 10 mL of prepared CS solution, and the mixture was stirred (~200 rpm) for about 30 min to reach a homogenous solution. The addition of Ag salts to the prepared homogeneous solution under stirring conditions resulted in the reduction of Ag ions. Prior to the loading of CUR into the fabricated CS-Ag nanohybrid, we dissolved CUR (2 mg/mL) in ethanol owing to its hydrophobic nature. For synthesizing the drug-loaded nanohybrid, dissolved CUR (0.25 mg/mL) was added to the prepared nanohybrid of CS-Ag under vigorous stirring conditions (~500 rpm for 15 min). Finally, the nanohybrids were left in liquid nitrogen for 5 min before transferring into the freeze-dryer.

### 2.4. Electrospinning and Crosslinking Setting

In order to fabricate a mixed fibrous structure, a dual pump electrospinning system (Fanavaran Nano Meghyas Ltd., Co., Tehran, Iran) was employed. This machine features two syringe pumps on both sides of the rotating collector drum. The homogenous nanohybrid of CS-Ag-CUR was placed into a 5 mL syringe, with a 21 gauge cut off tip. The applied voltage for the electrospinning of CS-Ag-CUR nanohybrid was 18 kW, injection flow rate 0.8 mL/h, and gap distance 16 cm. Before pouring the prepared SF solution into another 5 mL syringe, 40% (*v*/*v*) PEO was added to the solution in order to increase the SF electrospin ability. Then, the SF solution containing PEO was fed into another 5 mL syringe and converted to nanofibers under the electrospinning parameters of voltage of 20 kW, injection flow rate of 2.1 mL/h, and gap distance of 20 cm. The prepared SF nanofibers were treated with 75% ethanol vapor (5, 10, and 15 mL) around 20 min. In case of crosslinking CS-Ag-CUR nanofibers, the prepared nanofibers were transferred into a sealed chamber saturated with the vapor of GA solution (30, 40, and 50 mL). The nanofibers were treated with GA vapor for 24 h and heated and maintained at 120 °C for 24 h in an oven to remove the unreacted GA.

### 2.5. Characterization

Fourier Transform Infrared (FTIR) spectrophotometry was recorded on a Thermo AVATAR FT-IR spectrometer (Chicago, IL, USA). The crystalline structure of the samples was analyzed using X-ray diffraction (XRD, PHILIPS, PW1730, The Netherlands). The average size distribution and the surface charge of samples were obtained using Dynamic Light Scattering (DLS) and zeta potential measurements. Scanning Electron Microscopy (SEM) was also used to characterize the prepared samples’ morphology and diameter. The diameter of nanofibers was calculated using image analysis software (Digmizer, version 4.6.1, MedCalc software). The mechanical properties were studied using the testing machine (STM-20). Water contact angle measurements were carried out using the contact angle analyzer (OCA 15 plus, Dataphysics, Germany). To perform this test, a water droplet volume of 4 µL was used. Brookfield E230 was used for measuring viscosity.

### 2.6. Porosity

The porosity of samples was investigated by applying the liquid displacement procedure reported by Ju et al. [46]. Owing to quick permeation between samples without making any swellings or shrinking, ethanol was used as a displaced liquid. In the first stage, each sample (dry weight, W_0_) was immersed in a measuring cylinder containing a known volume (V_1_) of ethanol. The sample was kept in ethanol for 10 min. The total volume of the ethanol and the immersed sample was then recorded as V_2_. The immersed sample was removed from the measuring cylinder, and the residual volume of ethanol was registered as V_3_. The porosity was then measured by applying the equation below [46]:

(1) $$\mathrm{Porosity}\;(\%)=\frac{\left(V_{1}-V_{3}\right)}{\left(V_{2}-V_{3}\right)}\times 100$$

### 2.7. Swelling, Water Uptake, and Moisture Retention Test

Firstly, 20 mm × 20 mm of the prepared nanofiber was weighted. Next, the weighted nanofiber was immersed in 10 mL PBS (pH 7.7, 37 °C). At specific time intervals of 15, 30, 60, 90, 120, 150, and 180 min, the nanofiber was taken out and weighted after the surface water was absorbed using filter paper. The swelling ratio and water uptake were derived using equations 2 and 3, respectively [46].

(2) $$\mathrm{Swelling}\;\mathrm{ratio}\;(\%)=\frac{\left(\mathrm{Ws}-\mathrm{Wd}\right)}{\left(\mathrm{Wd}\right)}\times 100$$

(3) $$\mathrm{Water}\;\mathrm{uptake}\;(\%)=\frac{\left(\mathrm{Ws}-\mathrm{Wd}\right)}{\left(\mathrm{Ws}\right)}\times 100$$

where Ws denotes the weight of swollen nanofiber, and Wd represents the weight of dry nanofiber.

For the moisture retention test, first, the sample was saturated, taken out for centrifugation process at 500 rpm for 3 min, and then weighed precisely (Ws). Next, the sample was placed in an incubator with a constant temperature of 37 °C and relative humidity of 39 ± 1% and weighed every 30 min (Wt). The rate of water evaporation was measured employing the equation below [47]:

(4) $$\mathrm{Water}\;\mathrm{evaporation}\;\mathrm{rate}\;(\%)=\frac{\left(\mathrm{Ws}-\mathrm{Wt}\right)}{\left(\mathrm{Ws}-W0\right)}\times 100$$

### 2.8. Biodegradability

A biodegradability test was used in order to investigate the biodegradability behavior of the prepared nanofibers. First, 1 cm × 1 cm of the dry sample was weighed (W1), placed in PBS solution at pH 7.4 for 24 h, and then incubated at a physiological temperature of 37 °C. At specified time intervals of 0, 2, 4, 6, 8, 10, 12, and 14 days, the sample was brought out, washed with water several times, left in distilled water for 20 min to remove the nanofiber’s structure salts, and then dried. The proportion of mass loss or degradability was calculated based on the proportion of the remaining weight of the nanofibers employing the equation below:

(5) $$\mathrm{Remaining}\;\mathrm{weight}\;(\%)=\frac{\left(W1-W2\right)}{\left(W1\right)}\times 100$$

where W1 refers to the initial weight of the nanofiber before immersion in PBS solution, and W2 represents the final weight of the nanofiber after immersion in PBS solution.

### 2.9. CUR Entrapment and Loading Efficiency Measurement

Before adding ethyl acetate, 1 mg of lyophilized CS-Ag-CUR was dispersed in 1 mL of PBS. The ethyl acetate phase can be easily separated after being shaken. A UV–Vis spectrophotometer at 419 nm was used to measure the quantity of unbound CUR. Using Equations (6) and (7), the entrapment efficiency (EE) and loading efficiency (LE) of the drug were measured, respectively [48].

(6) $$\mathrm{EE}\;(\%)=\frac{\left(\mathrm{Total}\;\mathrm{quantity}\;\mathrm{of}\;\mathrm{CUR}\right)-\left(\mathrm{Free}\;\mathrm{quantity}\;\mathrm{of}\;\mathrm{CUR}\right)}{\left(\mathrm{Total}\;\mathrm{quantity}\;\mathrm{of}\;\mathrm{CUR}\right)}$$

(7) $$\mathrm{LE}\;(\%)=\frac{\left(\mathrm{Total}\;\mathrm{quantity}\;\mathrm{of}\;\mathrm{CUR}\right)-\left(\mathrm{Unbound}\;\mathrm{quantity}\;\mathrm{of}\;\mathrm{CUR}\right)}{\left(\mathrm{Total}\;\mathrm{quantity}\;\mathrm{of}\;\mathrm{Nanostructure}\right)}$$

### 2.10. Drug Release Assay

In order to identify and compare the release of CUR from SF-CS-Ag-CUR (CS 9% *w*/*v*) nanofiber at two pH of 5.4 and 7.4, we employed a dialysis technique. First, a piece of nanofibers (2 cm × 2 cm) was added to Spectrum-Labs dialysis bags (cut off Mw = 10–12 kDa). For the next 432 h, the dialysis bags containing the nanofibers mixture were submerged in 30 mL of two separate phosphate buffers with 20% *v*/*v* ethanol at 37 °C. We opted for phosphate buffer saline (PBS) (NaCl 0.138 M; KCl 0.0027 M) to dilute the lyophilized aptamer powders at pH 7.4. Regarding the adjustment of pH to 5.4, a pH meter and HCl solution were used. At specified time intervals, 300 µL of the medium was regularly separated for measuring the release of CUR within the buffer. A novel equal volume of fresh buffer was added to maintain a steady volume. In order to calculate absorption, the samples were evaluated spectrophotometrically at a 419 nm by a UV Vis spectrophotometer (UV-T60U; PG Instrument, Lutterworth, England) [49]. Equation (8) can be employed for calculating the percent of the discharged drug:

(8) $$\mathrm{CUR}\;\mathrm{released}\;(\%)=\frac{\left[\mathrm{CUR}\right]\mathrm{rel}}{\left[\mathrm{CUR}\right]\mathrm{load}}\;\text{×}\;100$$

where [CUR]load and [CUR]rel represent the amount of CUR entrapped throughout the nanofiber and the amount of CUR discharged from the nanofiber, respectively.

### 2.11. Antibacterial Study

In order to study the antibacterial activity of the samples against *E. coli* and *S. aureus* bacteria, the Minimum Inhibitory Concentration (MIC) test was used. Bacterial strains were cultured in Mueller hinton broth media for one day at 37 °C. Then, 0.5 McFarland solution was obtained by adding physiological serum with a volume ratio of 0.01 to the cultured bacteria. The optical density (OD_600_) was adjusted to 0.11. Each of the wells was filled with 100 µL of sterilized growth medium. Subsequently, 5 µL of bacteria suspensions were added to all wells except the last one. Next, 100 µL of sterilized SF-CS-Ag-CUR (CS 7% *w*/*v*) sample was introduced to the first well. Then, 100 µL of first well was separated and added to the second well. The process was repeated until well column 10 was reached. Well column 10 containing bacteria and culture medium was regarded as the positive group, and the 12th well containing culture medium was regarded as the negative group. Exactly the same procedure was repeated for the next sample. Finally, all of the plates were incubated at 37 °C for 24 h. The MIC for each row was determined employing ELISA Reader.

### 2.12. Cytotoxicity Analysis

In order to investigate the toxicity of SF-CS-Ag-CUR (CS 7% *w*/*v*) and SF-CS-Ag-CUR (CS 9% *w*/*v*) on the NIH 3T3 fibroblast cell line, MTT assays were employed. After adding the NIH 3T3 fibroblast cell line into a 24-well cell culture plate, the cells were incubated for 24 h at 37 °C and 5% CO2. Then, nanofibers were added to each well with their MIC concentration and incubated over 1, 3, and 7 days at 37 °C. The cells that were cultured without any treatment in DMEM basic medium including 10% FBS and 1% penicillin/streptomycin for 24 h were considered as the control group. Next, 50 μL of 5 mg/mL MTT solution was introduced to each well, and all wells were incubated for 4 h at 37 °C. The wells were rinsed with PBS buffer, filled with 150 µL of DMSO, and then stirred vigorously for 20 min in order to completely solubilize formazan. The optical density of each well was determined using an ELISA reader at the absorbance wavelength of 570 nm. For studying cell adhesion and morphology via SEM images, cells were fixed on the nanofibers through the following steps: First, the nanofibers were carefully removed from the incubator, each well culture medium was removed, and then 200 μL of GA was added to them. Second, GA was allowed to be in contact with the nanofibers for 3 h and then was moved away from the wells, after which the wells were washed with PBS solution. Finally, 200 μL of graded ethanol solution (50, 75, and 96%) was added to the wells for 10 min, and the nanofibers were separated from the wells, dried at ambient temperature, and finally photographed with SEM for cell adhesion assay.

## 3. Results

### 3.1. Morphological Characterization

**Figure 1:**
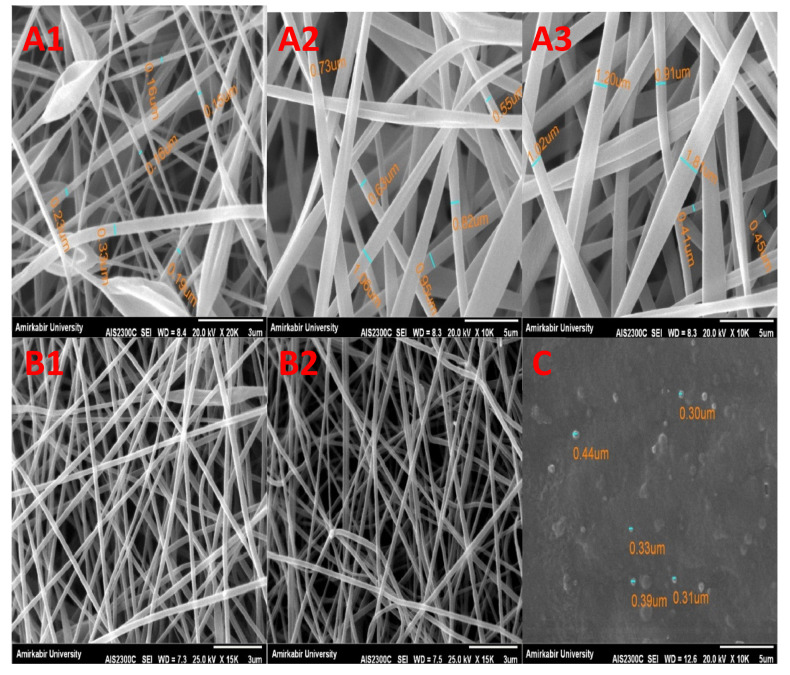
SEM images of SF-CS-Ag-CUR nanofibers at different CS concentration of (**A1**) 5% *w*/*v*, (**A2**) 7% *w*/*v*, and (**A3**) 9% *w*/*v*. SEM images of optimized SF-CS-Ag-CUR nanofibers before (**B1**) and- after crosslinking (**B2**) (CS-based nanofiber crosslinked with GA vapor, and SF nanofibers crosslinked with ethanol vapor). (**C**) SEM image of reduced Ag nanoparticles in CS solution.

**Figure 2:**
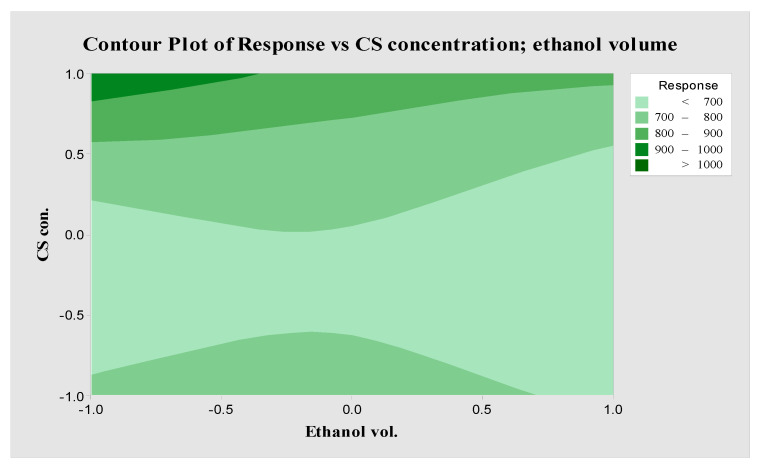
Contour plot for interaction between the concentration of CS and volume of ethanol on the diameter of nanofibers.

Among all the vital features of nanofibers employed for wound dressing, the diameter and morphological properties of nanofibers have been widely reported to be the most important ones. A central composite design (CCD) approach-based response surface methodology (RSM) was used for investigating and optimizing the effects of the concentration of CS hydrogel, ethanol volume in the crosslinking process of SF, and the concentration of GA as a cross-linker of CS hydrogel on the diameter and morphological properties of nanofibers. The experimental range and responses of SF-CS-Ag-CUR to the independent factors at three levels are presented in Table 1. Utilizing a multiple regression analysis of the obtained experimental data (Table 1), the relation between the diameter of SF-CS-Ag-CUR nanofibers and the test variables was represented based on the second-order polynomial equation. Values of *p* < 0.05 suggested that the model terms are significant. In this study, all the parameters except A, A^2^, and C were non-significant. Next, non-significant parameters were not considered, and the analysis was repeated again. According to the analysis of variance, all parameters have a p-value less than 0.05 (Table 2). F-value and *p*-value were used to interpret whether each of the parameters and their interactions were significant to be considered or not. As a result, the response and test variables were associated with the following second-order polynomial equation:

Diameter (nm) = 674.3 + (93.5 A) − (48.7 × C) + (1274 × A^2^) (9)

where A, B, and C refer to CS concentration, GA volume, and ethanol volume, respectively. Considering the coefficient of determination of 95.07%, the model is a good fit for the data. The results indicate that the GA volume does not cause any measurable change in the diameter of the nanofibers. The morphological analysis of SF-CS-Ag-CUR nanofibers at different CS concentrations is demonstrated in Figure 1(A1–A3). According to Figure 1(A1–A3) and Table 2, the increase or decrease in the concentration of CS causes a much greater change in the diameter of nanofibers as compared to other factors, suggesting the meaningful effect of the concentration of CS on nanofibers’ diameter and morphology. As a result, the concentration of CS is critical for the formation of beadles uniform nanofibers with diameters of 600–1100 nm. When CS was used in lower concentration, bead formation was increased, and the shape of the beads altered from spherical to elliptical-like in shape. This might be attributed to the increase in the solution’s viscosity. In fact, changes in polymer concentration from 5% to 9% (*w*/*v*) caused an increase in the amount of the solution viscosity from 13.4 to 58.1 cp. Figure 1(A1–A3) also showed that with an increase in the concentration of CS hydrogel, the density of nanofibers was also increased. The concentration of polymer used as a component in the electrospun fibrous wound dressing has a crucial impact on the viscosity of the solution. The interaction between chains gradually increases while the viscosity increases, favoring the formation of nanofibers without beads [50]. The combined effect of the concentrations of CS hydrogel and ethanol volume on the diameter of nanofibers is represented in Figure 2. The figure represents that the diameter of nanofibers increases with a decrease in ethanol volume as a cross-linker. Due to the reduced space between components in the fabricated nanofibers resulting from the increase in the cross-linker concentration, the nanofiber diameter decreases. On the other hand, the diameter decreases with a decrease in the concentration of CS hydrogel. This result is in accordance with the findings discussed by Zong et al. [51]. They found that higher conductivity resulted in the formation of beadles uniform nanofibers with reduced diameter. The maximum diameter of nanofibers (1021.3 nm) occurred at the CS concentration of 9% *w*/*v*, GA volume of 30 mL, and ethanol volume of 5 mL.

As wound dressing is a bioplatfrom that leads to high cell growth and proliferation, achieving a more intertwined structure can be helpful for wound healing. The SEM images of crosslinked and uncrosslinked SF-CS-Ag-CUR nanofibers at the optimized condition are shown in Figure 1(B1–B2). Crosslinking reactions caused the formation of physical entanglements between nanofibers. These interactions between nanofibers can maintain good cell adhesion, growth, and proliferation in the wound area. On the other hand, as displayed in Figure 1(A1–A3), both 7% and 9% (*w*/*v*) of CS concentrations were appropriate for the formation of smooth nanodimensional fibers. In this regard, nanofibers made of both 7% and 9% (*w*/*v*) of CS concentration with fixed GA and ethanol volumes of 30 and 5 mL, respectively, were opted for the mechanical and physical characterization of SF-CS-Ag-CUR nanofibers.

The morphological analysis of the reduced Ag nanoparticles in CS solutions is demonstrated in Figure 1C. Nanoparticles have a well-defined spherical shape. Uniformity in both size and shape is visible at a 5 μm scale, corroborating monodisperse nanoparticles at the nanoscale. The size distribution profile and poly disparity of the Ag nanoparticles were determined using DLS analysis. The size of synthesized Ag nanoparticles in CS solution is about 354 nm. This indicates proper antibacterial properties of synthesized Ag. In order to determine the stability of Ag nanoparticles, a zeta potential measurement was conducted. In general, zeta potential is a measure of the boundary between the stability and instability of a suspension. Nanoparticles with the same charge of either negative or positive in the suspension tend to repel each other and do not resist aggregation. A zeta potential of more than 30 mV or less than −30 mV confers the stability of particles. The synthesized nanoparticles of Ag by CS solution had a positive charge and acceptable potential for being stable (+31.1 mV).

### 3.2. Chemical Characterization

#### 3.2.1. FTIR

**Figure 3:**
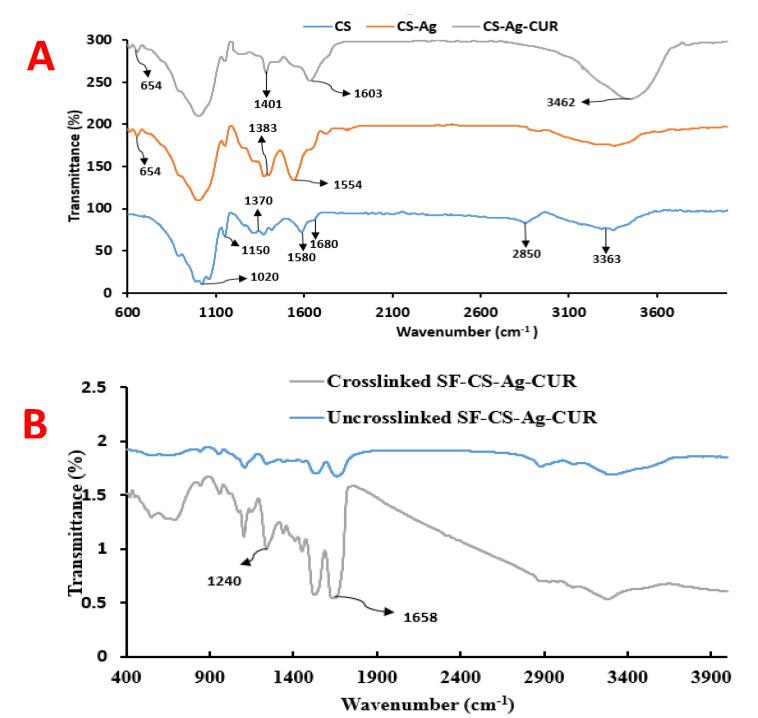
The FTIR spectra of (**A**) CS, CS-Ag, and CS-Ag-CUR (**B**) crosslinked SF-CS-Ag-CUR (CS 9% *w*/*v*) and uncrosslinked SF-CS-Ag-CUR (CS 9% *w*/*v*) nanofibers.

FTIR analysis was conducted in order to identify the presence of CS, Ag, and CUR. FTIR spectra for CS, CS-Ag, and CS-Ag-CUR are represented in Figure 3A. In the FTIR spectrum of CS, the band at 1020 cm^−1^ is characteristic peak of C-O bonding. The band at 1150 cm^−1^ is attributed to the asymmetric extension of the C-O-C bridge. The band at 1580 cm^−1^ is due to the N–H binding of amide I, corroborating the presence of acetylglucosamine unit of CS. The absorption band at around 1370 cm^−1^ can be ascribed to the CH_3_ symmetrical deformation [52]. Two bands at 1680 and 2850 cm^−1^ were detected due to the carbonyl stretching vibration of the secondary amide and C–H stretching, respectively. The wide band located in the region 3000–3560 cm^−1^ is related to N–H and O–H bonding, in agreement with previous studies [52,53].

The FTIR spectrum of CS-Ag were examined to validate the presence of Ag nanoparticles by new observed peaks. At 654 cm^−1^, the peak is referred to the presence of Ag nanoparticles [54]. All distinguishing bands of CS were detected in the FTIR spectrum of CS-Ag. The observed bands at 1580 and 1370 cm^−1^, which were ascribed to the N–H binding of amide I and CH3 symmetrical deformation, respectively, moved to another frequencies in the FTIR spectrum of CS-Ag and became broader. The band at 2850 cm^−1^ representing C–H stretching in the FTIR spectrum of CS disappeared in the FTIR spectrum of CS-Ag.

In the FTIR spectrum of CS-Ag-CUR, all distinguishing peaks of CS were observed. The band at 2850 cm^−1^ detected in the spectrum of CS was not detected in CS-Ag-CUR, signifying the complexation of CUR with other components. The band at 3363 cm^−1^ indicating N–H and O–H bonding in the FTIR spectrum of CS moved right and became broader. In addition, the presence of the peaks at 1603 and 1401 cm^−1^ might be owing to C=O stretching vibration of CUR.

The FTIR spectra of crosslinked and uncrosslinked SF-CS-Ag-CUR (CS 9% *w*/*v*) was shown in Figure 3B. In comparison with the FTIR spectrum of uncrosslinked SF-CS-Ag-CUR, the intensity of C-O and N–H corresponding peaks in the spectrum of SF-CS-Ag-CUR, ranging from 1625 to 1670 cm^−1^ and 1219–1245, respectively, increased, demonstrating successful crosslinking of the nanofiber and the formation of β-sheets.

#### 3.2.2. XRD

**Figure 4:**
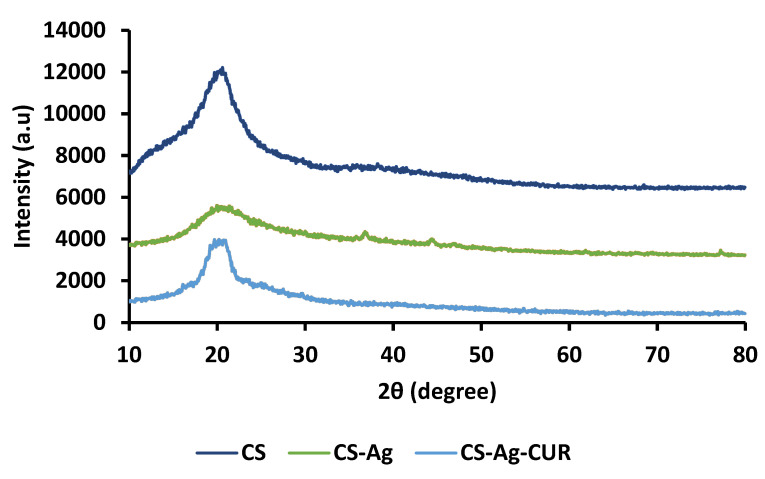
The XRD patterns of CS, CS-Ag, and CS-Ag-CUR.

XRD was performed to investigate the change in crystalline arrangement after incorporation of each component. The XRD patterns of CS, CS-Ag, CS-Ag-CUR nanostructures are shown in Figure 4. The diffractogram of CS indicated a peak at 2θ equal to 20.14°, corresponding to the (001) plane. In agreement with results reported by Rahmani et al. [53], this peak may be attributed to CS’s amorphous structure. In the XRD pattern of CS-Ag, it was found that the wide peak at 20.14°θ presented in CS has become broader and moved to the left, corroborating the presence of Ag nanoparticles in CS-Ag nanostructure. In addition, reduction in the intensity of broad peak of CS in the XRD pattern of CS-Ag may be attributed to the amorphous structure. The bands at 2θ = 38.1°, 44.4°, and 77.2° are characteristic peaks of Ag, confirming the incorporation of Ag nanoparticles in the CS-Ag [55]. The XRD of the CS-Ag-CUR nanostructure showed that the intensity of band at 2θ = 20.14° increased as compared to the XRD pattern of CS-Ag. This increase can be ascribed to the crystalline peak of CUR located between 5° and 30°, from which it can be inferred that the drug was successfully loaded into the CS-Ag [56].

#### 3.2.3. Mechanical Properties

**Figure 5:**
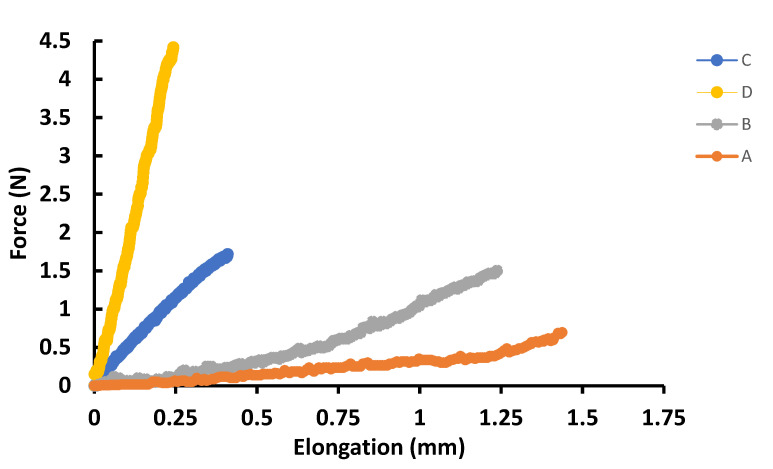
The mechanical analysis of (A) uncrosslinked SF-CS-Ag-CUR (CS 7% *w*/*v*), (B) crosslinked SF-CS-Ag-CUR (CS 7% *w*/*v*), (C) uncrosslinked SF-CS-Ag-CUR (CS 9% *w*/*v*), and (D) crosslinked SF-CS-Ag-CUR (CS 9% *w*/*v*) nanofibers.

The mechanical analysis of crosslinked and uncrosslinked SF-CS-Ag-CUR (CS 7% *w*/*v*) and SF-CS-Ag-CUR (CS 9% *w*/*v*) is demonstrated in Figure 5. Table 3 summarizes the mechanical properties of crosslinked and uncrosslinked SF-CS-Ag-CUR (CS 7% *w*/*v*) and SF-CS-Ag-CUR (CS 9% *w*/*v*) nanofibers. There was a significant difference between crosslinked and uncrosslinked samples. The crosslinked nanofibers with a higher amount of CS showed significantly higher tensile strength as compared to other groups, while uncrosslinked nanofibers with a lower amount of CS represented significantly higher strain. For both crosslinked and uncrosslinked nanofibers, a higher CS content in nanofibers leads to a higher tensile strength. Further, for both SF-CS-Ag-CUR (CS 7% *w*/*v*) and SF-CS-Ag-CUR (CS 9% *w*/*v*), uncrosslinked nanofibers show a higher strain. Considering all findings, we can conclude that interlocked structure of crosslinked nanofibers improves the durability of nanofibers against external force, which results in higher tensile strength. In contrast, in uncrosslinked nanofibers, the presence of weak and more flexible connections between components in the nanofibers leads to an increase in the tensile modulus and elongation. Increasing the concentration of the CS chain in the fabricate nanofibers will in fact lead to an increase in the viscosity and density, which then causes the nanofibers to become thicker and endure higher tensile strength while being stretched [57,58]. For those wound dressing and tissue engineering candidates considering the ideal platform for wound healing, the Young’s modulus must be between 2.9 and 150 MPa [59]. Considering the Young’s modulus of nanofibers from Table 3, we can conclude that all synthesized nanofibers can be considered as a potential candidate for wound healing.

### 3.3. Physical Characterization

#### 3.3.1. Porosity

The porosity of crosslinked and uncrosslinked SF-CS-Ag-CUR (CS 7% *w*/*v*) and SF-CS-Ag-CUR (CS 9% *w*/*v*) has been summarized in Table 3. The increase in the concentration of CS resulted in a decrease in the porosity of uncrosslinked nanofibers while considering a constant amount of GA as cross-linker. In fact, increased CS content directly correlated to increased diameter of the nanofibers and subsequently low surface area to volume ratio, leading toward a decrease in porosity. Contrary to uncrosslinked nanofibers, increase in the content of CS in crosslinked nanofibers caused an increase in porosity. This decrease can be ascribed to the reduction in the number of attaching points of amine and hydroxyl groups present in CS. In more detail, fierce competition between GA in finding available amine and hydroxyl groups as a result of the decrease in CS content caused the nanofibers’ structure to be compact with fewer pores [60].

#### 3.3.2. Water Uptake and Moisture Retention Analysis

**Figure 6:**
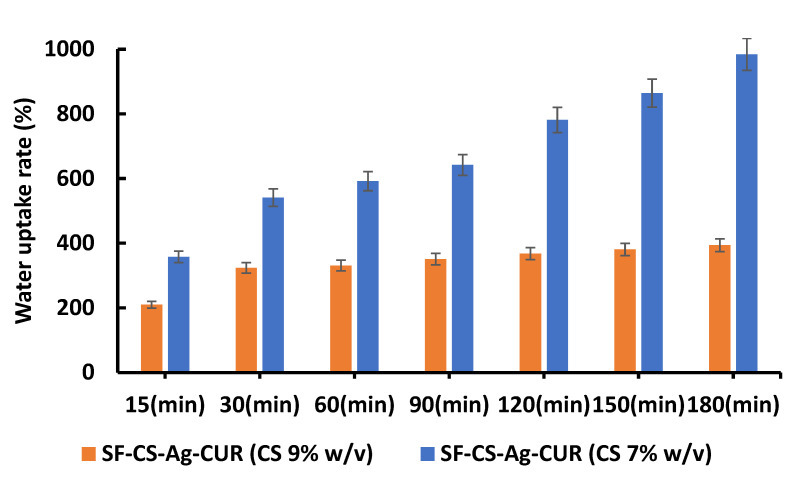
Water uptake profile of SF-CS-Ag-CUR (CS 7% *w*/*v*) and SF-CS-Ag-CUR (CS 9% *w*/*v*).

Among other factors, nutrient transport, cell signaling, and cell growth and proliferation are highly dependent on the water-holding capacity of a wound dressing and tissue engineering platform [61]. As Figure 6 depicts, the water-holding capacity of samples was characterized by an initial rapid increase in the first 30 min followed by a slower trend later in the process. The nanofibers holding less content of CS can hold more water between their nanostructures. As we consider the constant level of GA as a cross-linker, it might be ascribed to higher crosslinking in the structure of the nanofiber with less CS content. It was found that the water uptake capacity decreases with increased CS content in the composite. The water evaporation rate is considered another important factor in fabricating wound dressing. In fact, placing a wound dressing platform benefiting a low evaporation rate on the damaged area allows for an accelerated wound healing process by containing the existing moisture in the wound environment [62]. The water evaporation profiles were characterized by a rapid dehydration period in the first 240 min followed by a slow dehydration period. In fact, evaporation rates for SF-CS-Ag-CUR (CS 7% *w*/*v*) and SF-CS-Ag-CUR (CS 9% *w*/*v*) during 240 min were 75 and 76%, respectively. The occurrence of initial rapid dehydration might be ascribed to the evaporation of free water in the opening structure of CS chains. Additionally, the high concentration gradient of water could be the reason for the fast evaporation of water in the first 240 min. After initial rapid dehydration, the evaporation became slow. This sluggish evaporation can be ascribed to an interlocked structure, favoured by the presence of hydrophilic groups present in CS, i.e., amine and hydroxyl. Owing to higher crosslinking and a stronger interlocked structure, the nanofibers with less CS content caused a delay in evaporation. In fact, water molecules were entrapped effectively within the nanofibers with less CS content net structure, in line with the former results in the literature.

#### 3.3.3. Surface Wettability

**Figure 7:**
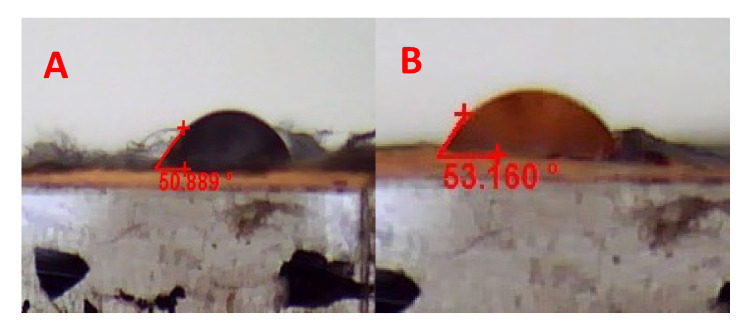
Water contact measurement of (**A**) SF-CS-Ag-CUR (CS 7% *w*/*v*) and (**B**) SF-CS-Ag-CUR (CS 9% *w*/*v*).

Water contact angles of SF-CS-Ag-CUR (CS 7% *w*/*v*) and SF-CS-Ag-CUR (CS 9% *w*/*v*) are shown in Figure 7. The water contact angle of SF-CS-Ag-CUR (CS 9% *w*/*v*) was 53.160, while the water contact angle for SF-CS-Ag-CUR (CS 7% *w*/*v*) was 50.889. These findings reveal the hydrophobic nature of CS hydrogel. Comparing the water contact angle of two groups, we can conclude that the fabricated nanofibers have suitable hydrophilicity, facilitating cell adhesion, growth, and proliferation, thus accelerating the wound healing process [63].

#### 3.3.4. Biodegradability

**Figure 8:**
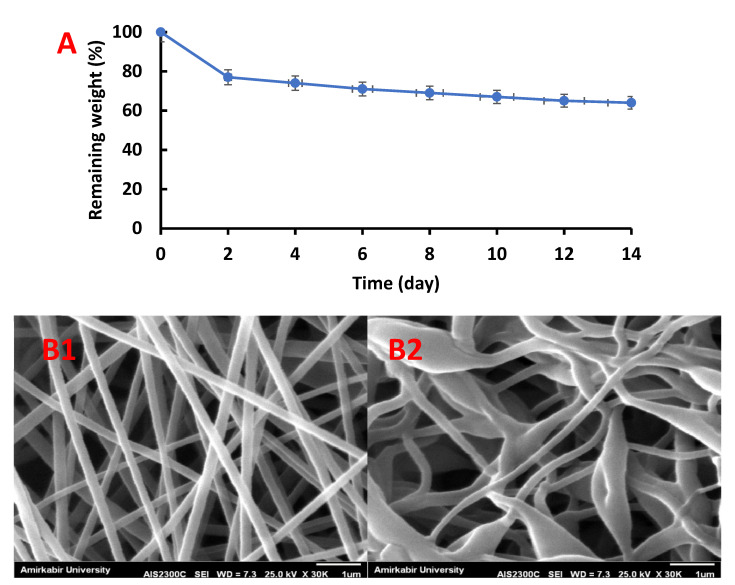
(**A**) Degradability of SF-CS-Ag-CUR (CS 9% *w*/*v*) and SEM images of of SF-CS-Ag-CUR (CS 9% *w*/*v*) nanofibers before (**B1**) and after 14 days of incubation (**B2**) in PBS solution.

The remaining weight of SF-CS-Ag-CUR (CS 9% *w*/*v*) nanofiber for every 2 days throughout 2 weeks of incubation in PBS solution adjusted to pH 7.4 at 37 °C has been shown in Figure 8A. The remaining weight of SF-CS-Ag-CUR (CS 9% *w*/*v*) nanofiber was 77% within 2 h, while the remaining weight of 64% was achieved for SF-CS-Ag-CUR (CS 9% *w*/*v*) nanofiber after 14 h. It was found that the degradation of CS occurs through the hydrogen bonding between residual amino and hydroxyl groups of CS and water molecules.

Further, the morphological characteristics of the nanofiber before and after 14 days of incubation in PBS solution were investigated using SEM images (Figure 8(B1,B2)). A comparison of SEM images of two groups of nanofibers showed that nanofibers incubated for two weeks were swollen. Interestingly, during the 14 days of incubation in PBS solution, no major changes in the surface morphology of the nanofibers were found. Considering several weeks to be the wound healing time, we can conclude that this platform could be a potential candidate for wound dressing.

### 3.4. CUR Loading and Entrapment Efficiency

The major problem associated with the administration of CUR is its poor solubility, causing a very poor bioavailability of 1 µg/mL [64,65]. Consequently, improving the loading and entrapment efficiencies of CUR can be regarded as a big step forward in the application of CUR in tissue engineering and wound dressing. After determining CUR’s free or unentrapped quantity in the ethyl acetate phase, Equations (6) and (7) can be considered for calculating loading and entrapment efficiencies of CUR, respectively. In order to investigate the effect of Ag nanoparticles in loading and entrapment efficiencies, we also measured loading and entrapment efficiencies for CS hydrogel (CS 9% *w*/*v*). The LE was measured 13% in CS-CUR (CS 9% *w*/*v*) and increased to 44% in CS-Ag-CUR (CS 9% *w*/*v*) (Table 4). This improvement in LE can be ascribed to the presence of Ag nanoparticles. In fact, amino and hydroxyl groups in CS chain and hydroxyl groups in CUR interact with Ag ions to form a high interpenetrated polymeric network. This interlocked structure results in the high drug entrapment, increasing the LE. Moreover, owing to the high surface area of Ag nanoparticles, the addition of Ag nanoparticles to the CS-CUR nanostructure provides more surface for the interaction of the drug and CS chains. The LE in the present study was higher than the CUR LE in pH-sensitive CS mesoporous silica nanoparticles [66], poly(ε-caprolactone) (PCL)-chitosan (CS)-CUR composite on which CUR-loaded CS was sprayed [67], and diblock copolymer micelles [68].

The EE of CUR in CS (CS 9% *w*/*v*) and CS-Ag (CS 9% *w*/*v*) was measured as 43% and 82%, respectively, similarly supporting the crucial impact of Ag nanoparticles in the increase in EE (Table 4). To overcome the poor water solubility and bioavailability of CUR, Peng et al. [69] prepared saponin-coated CUR nanoparticles. The prepared nanoparticles showed an encapsulation efficiency of 91%. In another study, Alizadeh et al. [68] successfully synthesized diblock copolymer micelles for the treatment of breast cancer. The CUR encapsulation efficiency in the prepared micelles was 64%. In addition, Kar et al. [70] fabricated montmorillonite clay as CUR carriers for targeting highly invasive FR-positive carcinomas. The encapsulation efficiency of CUR was reported to be 67%.

### 3.5. Release of CUR

**Figure 9:**
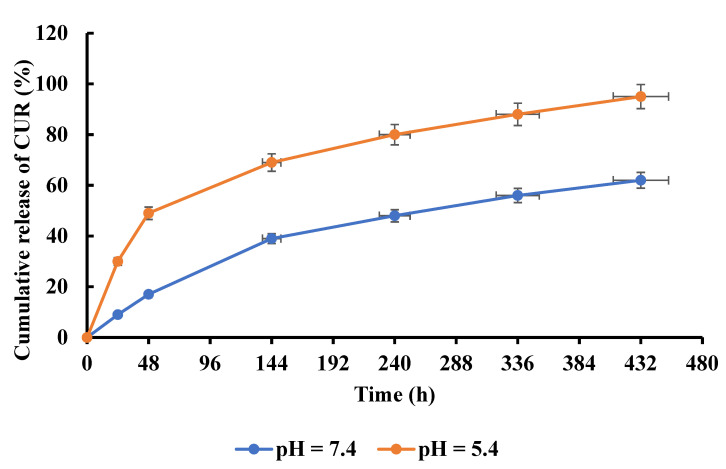
Cumulative release profile of CUR from SF-CS-Ag-CUR (CS 9% *w*/*v*) nanofiber at acidic and neutral environment.

We studied the release mechanism in order to confirm the pH-sensitive release of CUR from SF-CS-Ag-CUR (CS 9% *w*/*v*) nanofiber. As stated above, the release of CUR was investigated using the dialysis technique at pH = 7.4 and pH = 5.4 at 37 °C (the typical human body’s temperature) for 432 h (Figure 9). After passing 144 h, the cumulative release of CUR from SF-CS-Ag-CUR (CS 9% *w*/*v*) nanofiber was measured to be 39% at pH 7.4. In contrast, at pH = 5.4 (acidic conditions), the overall release of CUR from SF-CS-Ag-CUR (CS 9% *w*/*v*) nanofiber was 69% after 144 h. At pH 5.4, 95% of CUR was released after 432 h, while at pH 7.4, only 62% was released after 432 h. The pH-sensitive release from the fabricated SF-CS-Ag-CUR (CS 9% *w*/*v*) nanofiber can be attributed to the swelling characteristics of CS hydrogel. In fact, the repulsive force between the functional groups of the components in the nanofiber, i.e., the amino and hydroxyl groups in CS and hydroxyl group in CUR, can enhance the free spaces within components in acidic conditions, increasing the penetration of buffer into disintegrated nanofiber, resulting in the increased drug release from the SF-CS-Ag-CUR (CS 9% *w*/*v*) nanofiber at pH = 5.4 as compared to pH = 7.4. On the other hand, at a higher pH, the number of hydronium (H_3_O^+^) reduces. As a result, the CS chains failed to absorb water and swell, resulting in the formation of CS polymer chains accumulated around the nanofiber, functioning as a barrier to decrease the release of CUR from the nanofiber. Ahmadi Nasab et al. [66] prepared pH-responsive nanoparticles copped with CS for CUR delivery, which released about 28% of CUR at pH 5.5 after 24 h. CUR release from SF-CS-Ag-CUR (CS 9% *w*/*v*) nanofiber was 30% after 24 h at pH 5.4. In addition, the cumulative release of CUR from SF-CS-Ag-CUR nanofiber at pH 7.4 after 24 h was less than that of CUR reported by Ahmadi Nasab and colleagues–about 14% of CUR was discharged from the pH-responsive chitosan mesoporous silica nanoparticles after 24 h–while in the present work, only 9% of CUR was released at pH 7.4 after 24 h. Further, in research conducted by Fahimirad et al. [67], 34.56 and 30.78% of CUR were released from poly(ε-caprolactone) (PCL)-CS-CUR and PCL-CS-CUR sprayed with CUR-loaded CS nanoparticles (CURCSNPs) at pH 7.4 after 24 h, whereas in the present study, 39% of CUR was released from SF-CS-Ag-CUR (CS 9% *w*/*v*) after 144 h at pH 7.4.

### 3.6. Antibacterial Property

The antibacterial activity of SF-CS-Ag-CUR (CS 7% *w*/*v*) and SF-CS-Ag-CUR (CS 9% *w*/*v*) nanofibers against two *E. coli* (Gram-negative) and *S. aureus* (Gram-positive) bacteria was determined by employing the MIC method summarized in Table 5. Tetracycline was regarded as the control group in this experiment. Each test was replicated three times independently. The MIC result of SF-CS-Ag-CUR (CS 7% *w*/*v*) and SF-CS-Ag-CUR (CS 9% *w*/*v*) nanofibers against *E. coli* bacteria was 0.94 and 1.23 mg/mL, respectively. In the case of *S. aureus* bacteria, the quantity of SF-CS-Ag-CUR (CS 7% *w*/*v*) and SF-CS-Ag-CUR (CS 9% *w*/*v*) nanofibers for MIC was measured to be 1.04 and 1.36 mg/mL, respectively. The doses at which SF-CS-Ag-CUR (CS 7% *w*/*v*) nanofiber shows antibacterial activity against *E. coli* and *S. aureus* bacteria are the lowest, corroborating the excellent antibacterial property of SF-CS-Ag-CUR (CS 7% *w*/*v*) nanofiber. These findings correlate fairly well with those of Aliasghari et al. [71]. They concluded that an increase in bacterial growth resulted from a decrease in CS concentration.

### 3.7. Optical Density Measurement

**Figure 10:**
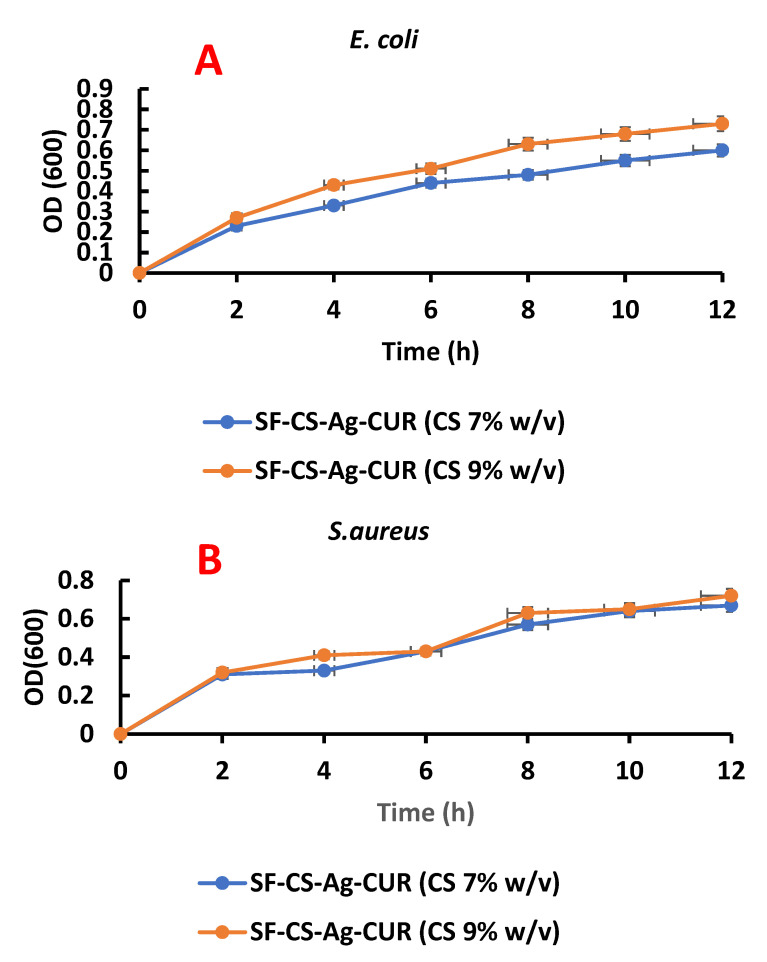
OD measurement of (**A**) *E. coli* and (**B**) *S. aureus* for MIC concentration of SF-CS-Ag-CUR (CS 7% *w*/*v*) and SF-CS-Ag-CUR (CS 9% *w*/*v*).

To further make a comparison between the antibacterial activity of SF-CS-Ag-CUR (CS 7% *w*/*v*) and SF-CS-Ag-CUR (CS 9% *w*/*v*) nanofibers against *E. coli* and *S. aureus*, we investigated the stage of growth by measuring the optical density at 600 (*OD_600_*). The growth curve of both *E. coli* and *S. aureus* bacteria in the presence of SF-CS-Ag-CUR (CS 7% *w*/*v*) and SF-CS-Ag-CUR (CS 9% w/v) nanofibers is represented in Figure 10. This test was carried out at MIC concentration for SF-CS-Ag-CUR (CS 7% *w*/*v*) and SF-CS-Ag-CUR (CS 9% *w*/*v*) nanofibers over 12 h, and 300 µL of samples were extracted at 2-h intervals. The SF-CS-Ag-CUR (CS 7% *w*/*v*) nanofiber caused more growth inhibition against both *E. coli* and *S. aureus* bacteria as compared to SF-CS-Ag-CUR (CS 9% *w*/*v*) nanofiber. This finding indicates the inverse effect of CS concentration on bacterial growth. All these findings are also in accordance with the results discussed in the antibacterial property section.

### 3.8. In Vitro Cytotoxicity Assay

**Figure 11:**
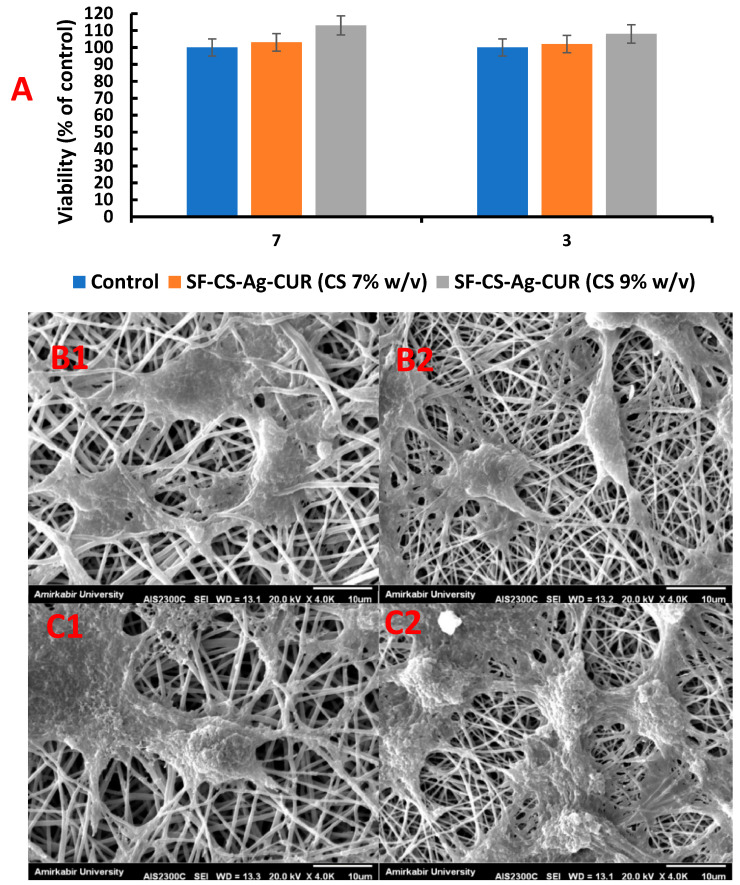
(**A**) MTT assay of seeded NIH 3T3 fibroblast cells on SF-CS-Ag-CUR (CS 7% *w*/*v*) and SF-CS-Ag-CUR (CS 9% *w*/*v*) and SEM images of NIH 3T3 fibroblast cells on SF-CS-Ag-CUR (CS 7% *w*/*v*) after 3 (**B1**) and 7 days (**B2**) of cell culture and on SF-CS-Ag-CUR (CS 9% *w*/*v*) after 3 (**C1**) and 7 days (**C2**) of cell culture.

MTT assay was used to determine the cytotoxicity effect of SF-CS-Ag-CUR (CS 7% *w*/*v*) and SF-CS-Ag-CUR (CS 9% *w*/*v*) nanofibers on NIH 3T3 fibroblast cells after the incubation of 1, 3, and 7 days and to find out if the nanofibers can be applied without showing toxicity. NIH 3T3 fibroblast cells received no treatment and were regarded as the control group. Cell viability higher than 80% is acceptable for considering a nanofiber as a biocompatible platform for wound dressing applications based on ISO standards. According to the results represented in Figure 11A, the cell viability for all designated days was higher than 80% for both of nanofibers, representing the biocompatibility of the nanofibers. On the other hand, MTT assay revealed that as the time of incubation of NIH 3T3 fibroblast cells with SF-CS-Ag-CUR (CS 7% *w*/*v*) and SF-CS-Ag-CUR (CS 9% *w*/*v*) nanofibers prolonged, the viability of cell increased subsequently. This result corroborated that the fabricated nanofibers do not have cytotoxic effects on cancer cells by themselves and only increase the growth and proliferation of fibroblast cells. The SEM images of SF-CS-Ag-CUR (CS 7% *w*/*v*) and SF-CS-Ag-CUR (CS 9% *w*/*v*) nanofibers after 3 and 7 days of incubation are represented in Figure 11B,C. The proliferation of NIH 3T3 fibroblast cells on both nanofibers increased as the time of incubation increased from 3 to 7 days. On the contrary, the adhesion and proliferation of NIH 3T3 fibroblast cells on SF-CS-Ag-CUR (CS 9% *w*/*v*) was much more than that of SF-CS-Ag-CUR (CS 7% *w*/*v*), suggesting the positive effect of CS on adhesion.

## 4. Conclusions

In the present study, a wound dressing composed of SF, CS, Ag, and CUR was fabricated via the electrospinning method. The developed nanofibers have the potential to concurrently proliferate the growth and spread of cells and release drugs in an acidic environment. A three-level-three-factor central composite design (CCD) approach-based response surface methodology (RSM) was applied to investigate the effect of the solution parameters on the nanofiber size and morphology. The results obtained indicated that the physical and mechanical properties of the nanofibers are dependent on the CS concentration and that CS hydrogel plays an important role in the pH-responsive release of CUR. The addition of Ag nanoparticles to the nanofibers results in a larger surface for interaction with the drug, hence leading to significantly higher CUR loading and entrapment into the fabricated nanofibers. As shown by MIC analysis on *E. coli* and *S. aureus*, CS concentration has noticeable effects on the antibacterial activities of both gram-positive and gram-negative bacteria. MTT assay demonstrated the biocompatibility and effectiveness of the designed nanofibers for NIH 3T3 fibroblast cell adhesion, growth, and proliferation. Thus, it is envisaged that the developed nanoplatform would be an ideal candidate for biomedical and healthcare applications as well as pharmaceutical science.

## Figures and Tables

**Table 1:** Matrix of CCD; factors and experimental responses.

No.	CS Concentration (A)	GA Volume (B)	Ethanol Volume (C)	(A) (*w*/*v*) %	(B) (mL)	(C) (mL)	Morphology	Mean Fiber Diameter (nm) ± Standard Deviation
1	−1	−1	−1	5	30	5	v	721.2 ± 2.7
2	1	−1	−1	9	50	5	v	1021.3 ± 2.7
3	−1	1	−1	5	30	5	v	739.2 ± 5.6
4	1	1	−1	9	50	5	i–v	943.1 ± 3.7
5	−1	−1	1	5	30	15	Fiber	692.9 ± 6.4
6	1	−1	1	9	50	15	i	826.3 ± 2.8
7	−1	1	1	5	30	15	Fiber	645.4 ± 4.4
8	1	1	1	9	50	15	i	817.7 ± 3.7
9	−1	0	0	5	30	10	fiber	742.4 ± 1.5
10	1	0	0	9	50	10	i	867.6 ± 8.4
11	0	−1	0	7	40	10	i	771.6 ± 8.7
12	0	1	0	7	40	10	Fiber	672.3 ± 4.1
13	0	0	−1	7	40	5	v	663.7 ± 3.6
14	0	0	1	7	40	15	Fiber	618.8 ± 1.1
15	0	0	0	7	40	10	fiber	645.1 ± 9.4

[i] Beads on CS nanofibers; [v] Beads on SF nanofibers.

**Table 2:** Analysis of variance of the model estimating the diameter of nanofibers.

Source	*p*-Value	F-Value
A	0	37.05
C	0.009	10.07
A^2^	0.001	22.94

**Table 3:** Mechanical and physical characteristics of prepared electrospun nanofibers.

Samples		Young’s Modulus (MPa)	Ultimate Tensile Stress (N)	Strain at Break (mm)	Porosity (%)	Water Contact Angle (°)
Crosslinked	SF-CS-Ag-CUR (CS 7% *w*/*v*)	7.87 ± 5.2	1.5 ± 7.6	1.24 ± 4.6	77 ± 6.2	50.889
Crosslinked	SF-CS-Ag-CUR (CS 9% *w*/*v*)	117.96 ± 2.4	4.42 ± 1.7	0.24 ± 3.2	84 ± 4.3	53.160
Un-crosslinked	SF-CS-Ag-CUR (CS 7% *w*/*v*)	3.14 ± 4.6	0.69 ± 2.3	1.44 ± 3.7	87 ± 3.2	
Un-crosslinked	SF-CS-Ag-CUR (CS 9% *w*/*v*)	27.18 ± 6.5	1.72 ± 6.9	0.41 ± 1.4	76 ± 4.9	

**Table 4:** Change in CUR LE and EE after the addition of CS.

	CS (CS 9% *w*/*v*)	CS-Ag (CS 9% *w*/*v*)	Change (%)
**LE (%)**	13	44	+31
**EE (%)**	43	82	39+

**Table 5:** MIC values of SF-CS-Ag-CUR (CS 7% *w*/*v*) and SF-CS-Ag-CUR (CS 9% *w*/*v*) nanofibers.

Bacteria	MIC (mg/mL)		
	SF-CS-Ag-CUR (CS 7% *w*/*v*)	SF-CS-Ag-CUR (CS 9% *w*/*v*)	Tetracycline
*S. aureus*	1.04 ± 5.1	1.36 ± 7.8	0.9 ± 3.4
*E. coli*	0.94 ± 3.6	1.23 ± 8.4	0.9 ± 4.7

## Data Availability

Data within this article are available on request.
